# ASSESSING BRAIN VOLUMETRICS IN THE AGING BRAIN USING COMPUTED TOMOGRAPHY

**DOI:** 10.1093/geroni/igz038.2180

**Published:** 2019-11-08

**Authors:** Andrei Irimia, Alexander Maher, Kenneth Rostowsky

**Affiliations:** University of Southern California, Los Angeles, California, United States

## Abstract

Although broadly utilized, computed tomography (CT) has been superseded by magnetic resonance imaging (MRI) for the volumetric assessment of white matter (WM), grey matter (GM) and cerebrospinal fluid (CSF) in the aging human brain. Nevertheless, many scenarios remain where MRI is unavailable or discouraged; furthermore, in developing countries, CT can often be the only accessible imaging method for assessing brain structure in older patients with mild cognitive impairment or Alzheimer’s disease. Thus, there is merit in developing effective approaches for the estimation of brain volumetrics using CT. Here, MRI and CT scans were acquired from 10 older adults [mean (µ) ± standard deviation (σ) of age = 65 ± 7 yrs; 5 females]. To demarcate WM, GM and CSF from head CT, we developed a brain segmentation method based upon probabilistic, atlas-dependent classification. MRI-only segmentation was compared to CT-only segmentation; similarity was calculated through the Dice coefficient (DC). A normal distribution of DCs was found after contrasting the methods [µ ± σ across participants: 85.5% ± 4.6% (WM), 86.7% ± 5.6% (GM) and 91.3% ± 2.8% (CSF)], suggesting a satisfactory capacity of CT to assess brain volumetrics. Sensitivity was adequate: WM, GM and CSF volumes were estimated within ~5%, ~4% and ~3% of their MRI-based values. There was no indication of volume over- or under-estimation with CT [t (9) = 0.89, p > 0.80]. These results facilitate the integration of CT-based brain volumetrics with MRI, thereby offering a wider range of methods for quantifying macroscale brain changes in neurodegenerative diseases.

